# A Rare Dual Diagnosis of Duodenal and Biliary Atresia in a Premature Infant

**DOI:** 10.7759/cureus.18218

**Published:** 2021-09-23

**Authors:** Andrew Hess, Christa Zino, Juan Camps

**Affiliations:** 1 Pediatric Surgery, Prisma Health Richland Hospital, Columbia, USA; 2 General Surgery, Grand Strand Medical Center, Myrtle Beach, USA

**Keywords:** duodenal atresia, biliary atresia, in vitro fertilization, premature infant, kasai procedure, duodeno-duodenostomy

## Abstract

We present a case of a baby conceived via in vitro fertilization and born prematurely with duodenal atresia. It was later discovered that the patient also had biliary atresia. Both defects were repaired surgically via duodeno-duodenostomy and Kasai procedures, respectively. Our case presents the rare event of both duodenal and biliary atresia as well as management in this case. Although this dual diagnosis is relatively uncommon, such a possibility should be considered in certain cases. This case also adds to the literature opening up further investigation for a likely link between these two anomalies.

## Introduction

In this case report, we will present the rare case of a premature infant diagnosed with both duodenal and biliary atresia. Although the incident of concurrent duodenal and biliary atresia is not well documented in current literature, there is a known association with prematurity [1]. Due to the sparse literature, it can be difficult to predict the prognosis of patients with both atretic events. The significance of this case is to walk through the diagnostic decision-making as well as increase documentation of simultaneous atretic events. The infant is currently progressing well but will likely require a liver transplant for long-term management.

## Case presentation

The patient’s birth history includes conception via in vitro fertilization of an anonymous donor egg (“22-year-old”) with a normal implantation genetic screen. Duodenal atresia was first suspected on a 25-week prenatal ultrasound that showed an anechoic area distal to the stomach (double bubble sign) suspicious for the narrow superior small intestine. Pregnancy was complicated by advanced maternal age, fibroid uterus, concern for duodenal atresia, significant anxiety, and mild polyhydramnios. The infant was born prematurely due to polyhydramnios secondary to duodenal atresia at 32 weeks 6 days by vaginal delivery, Apgar scores of 6 and 8, and birth weight of 1,509 grams (11th percentile), to a 43-year-old G1P0A0 mother. The infant’s Karyotype showed 46, XY with apparently normal chromosomes. Physical exam at birth showed no abnormalities. The abdomen was soft and flat with hypoactive bowel sounds and no hepatosplenomegaly. The skin was pink and well perfused. Echocardiogram on day of life (DOL) 1 showed possible small Secundum atrial septal defect with a small, restrictive, left-to-right patent ductus arteriosus. There was no evidence of pulmonary hypertension or abnormal left ventricular size, thickness, or function. 

An x-ray on DOL 0 confirmed duodenal atresia. On DOL 2, a successful duodeno-duodenostomy procedure with bypass of the duodenal web was completed. The gallbladder and pancreas were both visualized, and bile was found in the proximal portion of the duodenum. No bile was found in the distal portion. No other signs of atretic bowel were appreciated. The patient was extubated from the procedure on post-op day (POD) 3, and feeds were started POD 7 via an orogastric tube. On POD 5, there was an increasing concern for hyperbilirubinemia due to worsening cholestasis. This was thought to be related to post-op edema obstructing bile flow. Liver function tests showed normal enzymes and there was an initial decline in cholestasis followed by the bilirubin again rising. Ursodiol was started to correct elevated levels. An abdominal ultrasound, obtained to rule out biliary atresia, showed liver was normal in size contour and echogenicity without focal abnormalities. There was no intra or extrahepatic biliary dilatation, and although the gallbladder was not seen (likely due to bowel gas), the normal flow was appreciated. The pancreas was visualized and noted to be unremarkable. Total and direct bilirubin remained elevated (4.4/3.2) and the diet was modified to try and correct this. Hyperbilirubinemia did not correct (5.4/3.8), prior ultrasound was reviewed, and a hepatobiliary iminodiacetic acid (HIDA) scan was suggested to further investigate. HIDA scan on DOL 13 showed no significant biliary excretion, suggestive of obstruction of the common bile duct. A repeat ultrasound showed no evidence of gallbladder or biliary duct dilation. With those findings, a multidisciplinary decision was made to proceed with an MR cholangiogram without contrast. The cholangiogram showed no intra or extrahepatic biliary ductal dilatation and the common bile duct was not distinctly visualized. Additionally, no gallbladder was visualized. Due to consistent abnormal biliary tree results and persistent elevation of the infant's bilirubin, a Kasai with liver biopsy was performed on DOL 39. Intraoperative findings showed an atretic gallbladder and bile duct and we were unable to canulate for a cholangiogram. Formal extra-hepatic bile duct dissection was done, common bile duct and portal plate were fibrous tissue with no bile drainage. Formal wedge biopsy of the edge of the liver was done, biopsy showed giant cell hepatitis with bile ductular proliferation, cholestasis, and fibrous septae formation (stage 2/3 out of 4), and the common bile duct and gallbladder, excision showed fibrotic tissue with severely atretic bile duct. The patient tolerated surgery well and was able to resume oral feeds POD 5. Liver function tests showed bilirubin of total: 0.9 mg/dL, direct: 0.5 mg/dL, and indirect: 0.4 mg/dL, albumin of 3.4 g/dL, AST of 125 U/L, ALT of 119 U/L, and ALP of 398 ng/mL. The most recent liver ultrasound showed no visualization of bile ducts and no visualization of the gallbladder, but the otherwise normal liver size and blood flow. Due to the severity of the disease, a liver transplant is the likely next best step. A regular genetic screen was completed and showed no abnormalities.

## Discussion

Duodenal atresia occurs when the epithelial cells of the duodenal tract fail to recanalize or remain closed due to excessive endodermal proliferation. The exact etiology causing either of these two anomalies has yet to be determined [2]. The etiology of biliary atresia is also unknown. Currently, it is presumed to be the result of a neonatal inflammatory process of either autoimmune nature or secondary to viral infection. The currently proposed viruses thought to be involved are cytomegalovirus, rotavirus, and reovirus [3]. Duodenal and biliary atresia co-occurrence, while uncommon, is known to occur in Martinez-Frias Syndrome (MFS). MFS is reported to include pancreatic hypoplasia, intestinal atresias, extrahepatic biliary aplasia, or hypoplasia, with or without tracheoesophageal fistula, and sometimes associated with consanguinity [4]. We considered the possibility that our patient may have this syndrome, but because of several diagnostic criteria lacking, this syndrome is unlikely. Although biliary atresia with concomitant duodenal atresia is a rare occurrence, it is possible that there is an increased likelihood in premature infants [1]. About 25%-40% of patients with duodenal atresia are associated with trisomy 21, and 5% of patients with trisomy 21 have duodenal atresia [5]. There is also an increased incidence of cardiac anomalies in patients with duodenal atresia [6]. Both genetic testing and cardiac ultrasound in this patient were negative for abnormalities. While in vitro fertilization does appear to have an increased risk for some congenital anomalies, there is not a significant increase in anomalies of the gastrointestinal tract [7]. This indicates that the overall risk of duodenal and biliary atresia was increased due to prematurity as opposed to in vitro fertilization. With the egg being donated, there was an unknown past medical history.

For duodenal atresia, the best treatment option is to surgically intervene and remove the atretic portion of the duodenum, which was done in our patient on DOL 2. Biliary atresia does not have one best treatment plan, so several measures are done to determine the best treatment course. The extent of the disease varies in each case and with different portions of the biliary tree being affected more than others. The location of the damage is defined by the Ohm classification schemes and is currently one of the best measures of the amenability of the liver to drainage procedures and the prognostic indicators [2]. Type I involves atresia of the common bile duct, Type II the hepatic ducts, and Type III is atresia of the porta hepatis. These classifications are often not determined before surgical treatment and do not predict how successful the treatment will be [2]. Ursodiol can be given to facilitate bile flow through the liver during the post-operative period in patients with patent extrahepatic biliary systems [8]. Because the bilirubin levels in our patient were only moderately elevated it was unclear if there was true biliary atresia or just decreased flow through the biliary system. Therefore, we initially started Ursodiol to promote the patency of the biliary tree. When this therapy did not show improvement, further workup was completed which demonstrated true biliary atresia. The only two curative options for the treatment of biliary atresia are Kasai procedure and liver transplant [2]. The Kasai procedure involves transecting the small bowel and attaching the distal portion directly to the liver where the bile ductules exit to replace the atretic bile ducts. The proximal small bowel is then reconnected to the distal small bowel via a side-to-side anastomosis [9]. Kasai is often done first to prolong the need for liver transplants and is most beneficial for Type I. Although Types II and III are thought to be less amendable to Kasai drainage, all types are recommended to undergo surgery because improvement after surgery has been seen in all types [2].

## Conclusions

Concurrent duodenal and biliary atresia, outside the realms of a syndrome, appear to be closely related to prematurity although known cases of such occurrence are not well documented. Further understanding of the etiology of both atretic events may uncover a hidden link between gestational age and coinciding duodenal and biliary atresia. Additionally, the treatment options for coinciding duodenal and biliary atresia remain the same as one atretic event alone.
